# Gene expression profiles in the rat streptococcal cell wall-induced arthritis model identified using microarray analysis

**DOI:** 10.1186/ar1458

**Published:** 2004-11-19

**Authors:** Inmaculada Rioja, Chris L Clayton, Simon J Graham, Paul F Life, Marion C Dickson

**Affiliations:** 1 Rheumatoid Arthritis Disease Biology Department, GlaxoSmithKline, Medicines Research Centre, Stevenage, UK; 2 Transcriptome Analysis Department, GlaxoSmithKline, Medicines Research Centre, Stevenage, UK

**Keywords:** arthritis, differential gene expression, microarray, rat, SCW induced arthritis

## Abstract

Experimental arthritis models are considered valuable tools for delineating mechanisms of inflammation and autoimmune phenomena. Use of microarray-based methods represents a new and challenging approach that allows molecular dissection of complex autoimmune diseases such as arthritis. In order to characterize the temporal gene expression profile in joints from the reactivation model of streptococcal cell wall (SCW)-induced arthritis in Lewis (LEW/N) rats, total RNA was extracted from ankle joints from naïve, SCW injected, or phosphate buffered saline injected animals (time course study) and gene expression was analyzed using Affymetrix oligonucleotide microarray technology (RAE230A). After normalization and statistical analysis of data, 631 differentially expressed genes were sorted into clusters based on their levels and kinetics of expression using Spotfire^® ^profile search and K-mean cluster analysis. Microarray-based data for a subset of genes were validated using real-time PCR TaqMan^® ^analysis. Analysis of the microarray data identified 631 genes (441 upregulated and 190 downregulated) that were differentially expressed (Delta > 1.8, *P *< 0.01), showing specific levels and patterns of gene expression. The genes exhibiting the highest fold increase in expression on days -13.8, -13, or 3 were involved in chemotaxis, inflammatory response, cell adhesion and extracellular matrix remodelling. Transcriptome analysis identified 10 upregulated genes (Delta > 5), which have not previously been associated with arthritis pathology and are located in genomic regions associated with autoimmune disease. The majority of the downregulated genes were associated with metabolism, transport and regulation of muscle development. In conclusion, the present study describes the temporal expression of multiple disease-associated genes with potential pathophysiological roles in the reactivation model of SCW-induced arthritis in Lewis (LEW/N) rat. These findings improve our understanding of the molecular events that underlie the pathology in this animal model, which is potentially a valuable comparator to human rheumatoid arthritis (RA).

## Introduction

Rheumatoid arthritis (RA) is an autoimmune chronic inflammatory disease of unknown aetiology that is characterized by infiltration of monocytes, T cells and polymorphonuclear cells into the synovial joints. The pathogenesis of this disease is still poorly understood, and fundamental questions regarding the precise molecular nature and biological significance of the inflammatory changes remain to be answered [1,2]. A powerful way to gain insight into the molecular complexity and pathogenesis of arthritis has arisen from oligonucleotide-based microarray technology [3], because this platform provides an opportunity to analyze simultaneously the expression of a large number of genes in disease tissues.

The earliest preclinical stages of human RA are not easily accessible to investigation, but a diverse range of experimental arthritis models are considered valuable tools for delineating mechanisms of inflammation and autoimmune phenomena. An animal model that shares some of the hallmarks of human RA is the reactivation model of streptococcal cell wall (SCW)-induced arthritis in rats. In this model, a synovitis with maximal swelling at 24 hours is induced by local injection of SCW antigen directly into an ankle joint. The initial response is reactivated by systemic (intravenous) challenge with SCW, which produces a more prolonged and severe inflammation confined to the joint previously injected with SCW. In contrast to some other animal models, in which the arthritic response develops gradually and unpredictably, in this model the flare response develops synchronously, allowing precise analysis of pathophysiological mechanisms [4,5].

Some pathological changes observed in SCW-induced arthritis that are of relevance to human RA include infiltration of polymorphonuclear cells, CD4^+ ^T cells and macrophages, hyperplasia of the synovial lining layer, pannus formation and moderate erosion of cartilage and bone [4]. Previous reports have shown the dependency of this model on tumour necrosis factor (TNF)-α, IL-1α, IL-4, P-selectin, vascular cell adhesion molecule-1, macrophage inflammatory protein (MIP)-2, MIP-1α and monocyte chemoattractant protein (MCP)-1 [6,7]. Although the involvement of nitric oxide synthase (NOS) [8] and cyclo-oxygenase [9] in the development of SCW-induced arthritis has also been noted, a global analysis of coordinated gene expression during the time course of disease in this experimental arthritis model has not been investigated.

Arthritis involves many cell types from tissues adjacent to the synovium. Therefore, as shown in previous studies [10,11], analysis of gene expression profiles by processing whole homogenized joints can provide useful information about dysregulated genes, not only in synoviocytes but also in other, neighbouring cells (myocytes, osteocytes and chondrocytes) that may also contribute to disease pathology.

In the present study, whole homogenized rat ankle joints from naïve, SCW-injected and phosphate-buffered saline (PBS; vehicle)-injected animals, included in a time-course study, were analyzed for differential gene expression using the RAE230A Affymetrix GeneChip^® ^microarray (Affymetrix Inc., Santa Clara, CA, USA). In order to identify different patterns of gene expression during the course of SCW-induced arthritis, a selected set of genes whose expression was statistically significantly different between arthritic and control animals on days -13.8, -13 and 3 were analyzed using agglomerative hierarchical clustering, Spotfire^® ^(Spotfire Inc., Cambridge, MA, USA) profile search and K-means cluster analysis. Validation of microarray data for a subset of genes was performed by real-time RT-PCR TaqMan^® ^(Applied Biosystems, Foster City, CA, USA) analysis, which provides a highly accurate method for quantifying mRNA expression levels for any particular differentially expressed gene. To further investigate the possible association of 20 selected upregulated genes with arthritis pathogenesis, their chromosomal locations and the chromosomal locations of their corresponding human orthologue were compared with the locations of previously reported quantitative trait loci (QTLs) for inflammation, arthritis and other autoimmune diseases. Our findings show, for the first time, the gene expression profiles and kinetics of expression of hundreds of genes that are differentially expressed in arthritic joints from the reactivation model of SCW-induced arthritis in Lewis (LEW/N) rat, thereby improving our understanding of the biological pathways that contribute to the pathogenesis of arthritis in this animal model and providing a valuable comparator to human RA.

## Methods

### Reagents

The peptidoglycan–polysaccharide (PG-PS) 100p fraction of SCW was purchased from Lee Laboratories (Grayson, GA, USA). RAE230A Affymetrix GeneChip^® ^were purchased from Affymetrix Inc. All reagents required for RT-PCR were from PE Applied Biosystems (Warrington, UK). Forward and reverse primers were purchased from Invitrogen™ Life Technologies (Invitrogen Ltd, Paisley, UK). TaqMan^® ^probes were synthesized by PE Applied Biosystems. RiboGreen, used to quantify RNA, was obtained from Molecular Probes Inc. (Leiden, The Netherlands) and RNA 6000 Nano LabChip Kit^®^, used to assess RNA integrity, was from Agilent Technologies Inc. (Stockport, UK).

### Animals

All *in vivo *studies were undertaken in certified, dedicated *in vivo *experimental laboratories at the GlaxoSmithKline Medicines Research Centre (Stevenage, UK). The studies complied with national legislation and with local policies on the care and use of animals, and with related codes of practice. Male Lewis (LEW/N) rats obtained from Harlan UK Ltd (Oxon, UK), at age 6–7 weeks, were housed under standard conditions and received food and water *ad libitum*. Animals were habituated to the holding room for a minimum of 1 week before the experimental procedures.

### Induction and assessment of SCW-induced arthritis

SCW arthritis was induced in 6- to 8-week-old male Lewis (LEW/N) rats (weight 125–150 g) following a method similar to that previously described by Esser and coworkers [4]. A SCW preparation (PG-PS, 100p fraction) was suspended in PBS and 10 μl of the suspension containing 5 μg PG-PS from *Streptococcus pyogenes *was injected into the right ankle joint (day -14). Animals from control groups were injected similarly with 10 μl PBS. A group of noninjected rats was also included in our study to assess gene expression profiles in joints from naïve animals. Reactivation of the arthritic inflammation was induced 14 days after intra-articular injection (designated day 0) by intravenous injection of 200 μg PG-PS. This resulted in monoarticular arthritis involving the joint originally injected with PG-PS [7]. Ankle swelling at different time points was measured using a caliper. The inflammatory response is expressed as the change in ankle diameter relative to the starting diameter. Five animals injected with PG-PS or PBS were killed at different time points (4 hours after intra-articular injection [day -13.8], day -13, day -10, day 0, 6 hours after intravenous challenge [day 0.4], day 1, day 3 and day 7) and ankle joints were dissected, snap frozen in liquid nitrogen and stored at -80°C for subsequent analysis.

### Total RNA isolation from rat joints

Frozen ankle joints were pulverized in liquid nitrogen using a mortar and pestle, and total RNA was isolated from individual homogenized joints (four or five animals/group) using RNeasy^® ^Mini-kits (Qiagen Ltd, Crawley, UK), following the manufacturer's instructions. In our experimental design, a nonpooling strategy for total RNA samples was used (a total of 75 samples from different animals were analyzed). In order to ensure that no contamination with genomic DNA occurred, samples were treated for 15 min with 10 units of RNase-free DNase (Qiagen Ltd) at room temperature. RiboGreen^® ^RNA Quantitation Kit (Molecular Probes Inc.) with optical densities at 260 nm and 280 nm was used to determine the total RNA concentration of the samples. The quality of the RNA was assessed based on demonstration of distinct intact 28S and 18S ribosomal RNA bands using RNA 6000 Nano LabChip Kit^® ^(Agilent 2100 Bioanalyser; Agilent Technologies UK Ltd, Stockport, UK). Five of the 75 total RNA samples exhibited evidence of RNA degradation and were excluded from subsequent analyses.

### Oligonucleotide microarray analysis

The rat RAE230A GeneChip^® ^oligonucleotide microarray (Affymetrix Inc.), containing about 16,000 probe sets, representing 4699 well annotated full-length genes, 10,467 expressed sequence tags (ESTs) and 700 non-ESTs (excluding full lengths), was used to analyze gene expression profiles in joints from SCW-injected or PBS-injected animals during the course of disease. Isolated total RNA (10 μg/chip) was used to generate biotin-labelled cRNA. Aliquots of each sample (*n *= 70) were then hybridized to RAE230A Affymetrix GeneChip^® ^arrays at 45°C for 16 hours, followed by washing and staining, in accordance with the standard protocol described in the *Affymetrix GeneChip*^® ^*Expression Analysis Technical Manual *[12]. The GeneChip^®^s were scanned using the Affymetrix 3000 Scanner™ and the expression levels were calculated for all 16,000 probe sets (about 12,000 genes) with Affymetrix^® ^MicroArraySuite software (MAS 5.0).

### Statistical analysis of microarray data

The Affymetrix GeneChip^® ^data were processed, normalized and statistically analyzed (analysis of variance [ANOVA]) using Rosetta Resolver^® ^v3.2 software (Rosetta Biosoftware, Kirkland, WA, USA). Genes with *P *< 0.01 (ANOVA) were considered to be differentially expressed. Fold changes in gene expression were calculated by dividing the mean intensity signal from all the individual SCW-injected rats included in each group by the mean intensity signal from the corresponding PBS control group. The level of statistical significance was determined by ANOVA. Subsequent data analysis involved two-dimensional data visualization, principal component analysis (PCA) using SIMCA-P v10.2 Statistical Analysis Software (Umetrics, Windsor, UK) [13] and agglomerative hierarchical clustering analysis [14]. For identification of different temporal patterns and levels of gene expression, Spotfire^® ^profile search analysis and K-means clustering analysis [15] were performed using the Spotfire^® ^DecisionSite for Functional Genomics programme. In this analysis the mean signal intensity of gene expression in each group included in the study (four to five samples/group) was used. Identification of the ontology, accession number and chromosomal location of the genes of interest was performed combining information from GlaxoSmithKline Bioinformatics Databases and other existing public databases . The mapping of the differentially expressed genes to QTLs for arthritis was investigated using Rat and Human Genome browsers from Ensembl , Rat Genome Database  and the ARB Rat Genetic Database .

### Quantitative real-time PCR (TaqMan^®^)

Expression levels of selected genes found to be upregulated by gene array analysis were validated by real-time RT-PCR TaqMan^® ^analysis using the ABI Prism 7900 Sequence Detector System^® ^(PE Applied Biosystems, Foster City, CA, USA), as previously described [16]. For cDNA synthesis 600 ng total RNA (from the same samples analysed by RAE230A GeneChip^® ^microarray) were reverse transcribed using TaqMan^® ^RT reagents (PE Applied Biosystems) in a MJ Research PTC-200 PCR Peltier Thermal Cycler (MJ Research, Rayne Brauntree, Essex, UK).

TaqMan^® ^probes and primers for the genes of interest were designed using primer design software Primer Express™ (PE Applied Biosystems) and optimized for use. The forward primers, reverse primers and probes used are summarized in Table 1. The final optimized concentrations of forward primer, reverse primer and probe for all of the target genes were 900 nmol/l, 900 nmol/l and 100 nmol/l, respectively, except for CD14, for which the concentrations were 300 nmol/l, 300 nmol/l and 100 nmol/l. Standard curves for each individual target amplicon were constructed using sheared rat genomic DNA (BD Biosciences, Cowley, Oxford, UK). All PCR assays were performed in duplicate, and results are represented by the mean values of copy no./50 ng cDNA. Ubiquitin [17] was used as a housekeeping gene against which all samples were normalized.

### Data presentation

The data included in Table 2 show the mean fold change (Delta) increase or decrease in gene expression in joints from SWC-injected rats compared with the expression in the corresponding PBS control group, along with the *P *value. As selection criteria to present the most relevant genes, a cutoff of 1.8-fold increased/decreased expression and *P *< 0.01 were arbitrarily chosen. Gene expression profile plots (Fig. 6) represent data from Affymetrix Rat Genome RAE230A GeneChip^® ^and real-time RT-PCR TaqMan^® ^as the mean of signal intensity or the mean of normalized copy no./50 ng cDNA for all the samples from the same group (four to five), respectively.

## Results

### Time course of inflammation in the SCW-induced arthritis model

**Figure 1:**
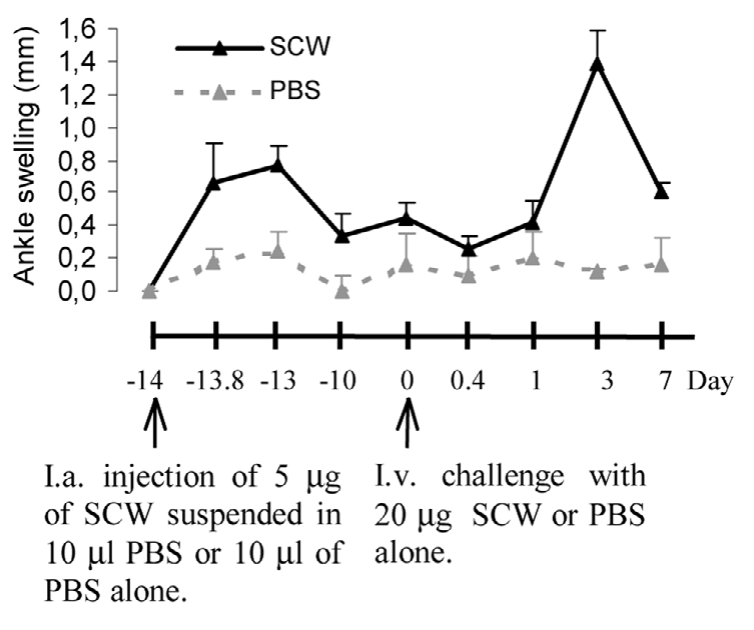
Schematic representation of the experimental design for the time course study in the reactivation model of streptococcal cell wall (SCW)-induced arthritis in Lewis (LEW/N) rats. The inflammatory response is represented as the change in ankle diameter (mm) relative to the starting diameter. Data are expressed as means ± standard error (four to five animals/group). Intra-articular (i.a.) injection of SCW resulted in increased ankle swelling that peaked 24 hours after injection (day -13) followed by a gradual reduction by day 0. At this time point, intravenous (i.v.) challenge with SCW led to reactivation of the inflammatory response, which peaked 72 hours thereafter (day 3). Animals injected with a suspension of SCW (continuous line) in PBS or with PBS alone (dashed line; five animals/group) were killed on the days indicated, and joints taken and processed for gene expression profiling analysis and mRNA quantification by GeneChip^® ^microarray and real-time RT-PCR TaqMan^®^, respectively. A group of naïve noninjected animals (*n *= 4) was also included in the study to assess basal expression levels of the analyzed genes.

Intra-articular injection of SCW resulted in increased ankle swelling that peaked 24 hours after injection (day -13), followed by a gradual reduction by day 0 (Fig. 1). At this time point intravenous challenge with SCW led to reactivation of the inflammatory response, which peaked 72 hours thereafter (day 3). Animals injected intra-articularly with PBS (vehicle in which the SCW was suspended) were used as control groups at each specific time point. Another group of naïve animals (noninjected rats) was used to assess a possible inflammatory response due to the intra-articular injection alone.

### Gene expression profiling in SCW-induced arthritis

**Figure 2:**
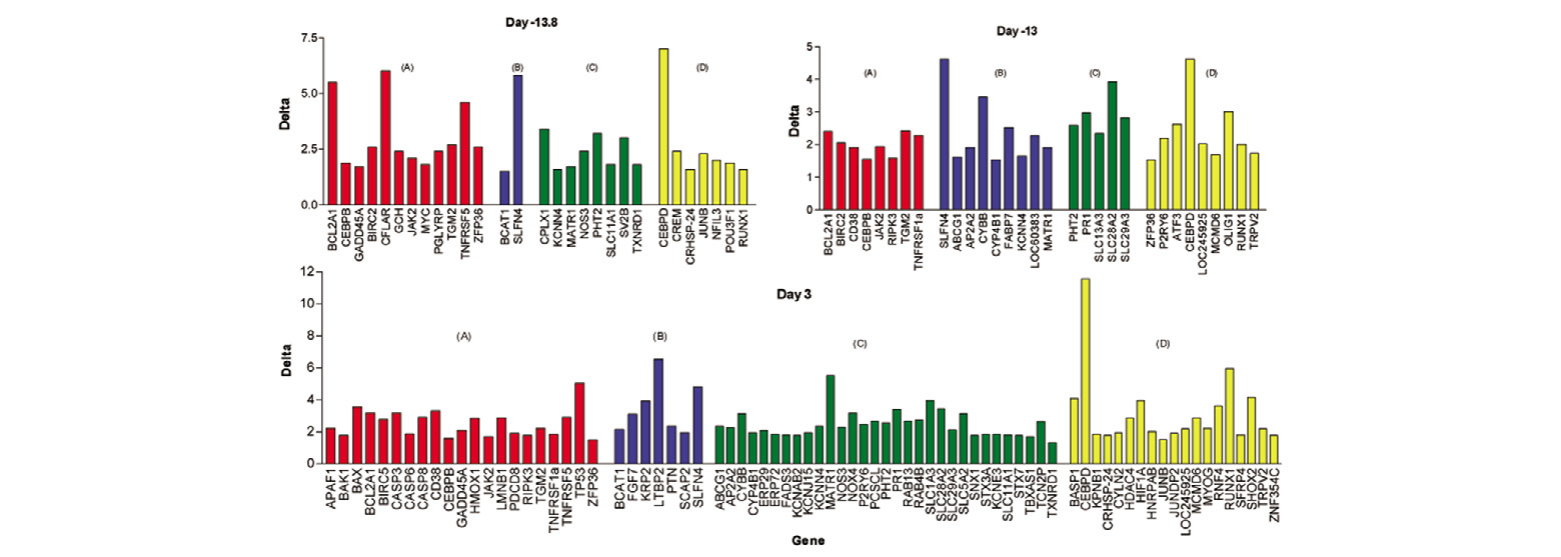
Representative graph of genes that were upregulated (Delta > 1.8 and *P *< 0.01) in arthritic joints from streptococcal cell wall (SCW)-induced arthritis model on day -13.8 (4 hours after systemic challenge), day -13 and day 3. The graphs represent the fold increase in gene expression (Delta) and the name of the genes associated with the following ontologies: apoptosis (A; red bars), regulation of cell cycle and cell proliferation (B; blue bars), transport (C; green bars) and regulation of transcription, DNA-dependent (D; yellow bars).

**Figure 3:**
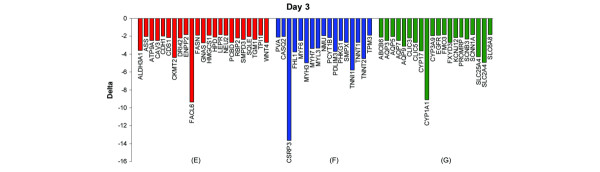
Downregulated genes (Delta < -1.8 and *P *< 0.01) in arthritic joints from streptococcal cell wall (SCW)-induced arthritis model on day 3 after systemic challenge. This graph shows the fold decrease in gene expression (Delta) on day 3 and the name of the downregulated genes associated with the following ontologies: metabolism (E; red bars), regulation of muscle development (F; blue bars) and transport (G; green bars).

Analysis of RAE230A GeneChip^® ^microarray data identified about 9000 probes (5479 upregulated and 3898 downregulated) that were differentially expressed to a highly significant degree (*P *< 0.01) in arthritic rat joints from the time course study. After applying selection criteria (Delta > 1.8 and *P *< 0.01), 631 of the dysregulated probes had well characterized full-length sequences in databases (441 upregulated and 190 downregulated) and 697 were unknown (ESTs; 444 upregulated and 253 downregulated). These genes are too numerous to describe in detail, and therefore we present a selected list of upregulated genes in Table 2 and Fig. 2, and a selection of downregulated genes based on the ontologies that reflect the major changes occurring in arthritic animals (Fig. 3). ESTs were excluded from Table 2 and from subsequent clustering analysis. See Additional file 1, which contains all genes that were upregulated and downregulated.

### Principal component analysis and hierarchical clustering

**Figure 4:**
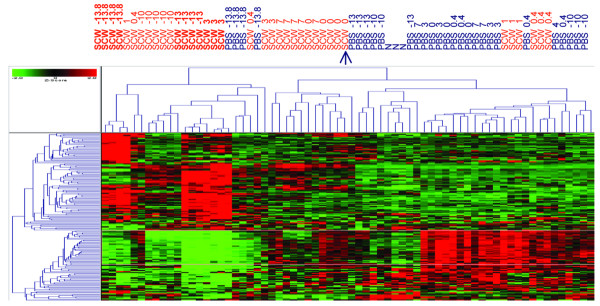
Heat map diagram of differential gene expression in joints from the time course study in the streptococcal cell wall (SCW)-induced arthritis in Lewis (LEW/N) rat. Gene expression data were obtained using Affymetrix Rat Genome RAE230A GeneChip^®^. The cluster diagram represents 631 differentially expressed probes with *P *< 0.01 and Delta > 1.8. Each column represents a single joint tissue and each row represents a single gene. Expression levels are coloured green for low intensities and red for high intensities (see scale at the top left corner). At the top of the cluster diagram is an enlarged panel including the names and hierarchical clustering order of the individual samples analyzed. Red names are joint tissues from SCW-injected animals, indicating the corresponding time point of sample collection, and blue names are the samples from the phosphate-buffered saline (PBS) control groups. As shown, the major changes in gene expression occurred in samples corresponding to arthritic animals from days -13.8 (4 hours after intra-articular injection of SCW), -13 and 3. N, naïve animals.

An overview of the experimental RAE230A GeneChip^® ^data was obtained using PCA (graphs not shown) [13] and agglomerative hierarchical clustering [14]. Both two-dimensional analyses identified day -13.8 (4 hours after intra-articular injection of SCW), day -13 and day 3 as the time points at which the greatest changes in gene expression in arthritic joints occurred in comparison with corresponding PBS control groups. The results from the hierarchical clustering are shown for visual inspection as a coloured heat map in Fig. 4. As shown on the x-axis (panel at the top of Fig. 4), the majority of the PBS samples clustered together, except the PBS samples from day -13.8, which clustered close to the SCW-injected animals from day 3. This observation indicated the presence of a mild inflammatory response in joints from rats killed 4 hours after the initial intra-articular injection of PBS, when compared with expression levels in joints from naïve animals or the PBS samples from later time points.

PCA and hierarchical clustering analysis allowed us to identify two outliers corresponding to arthritic animals from day 3, which did not show any sign of measurable inflammation after intravenous challenge. Both samples were excluded from subsequent mean or Delta calculations.

### Identification of different patterns of gene expression

**Figure 5:**
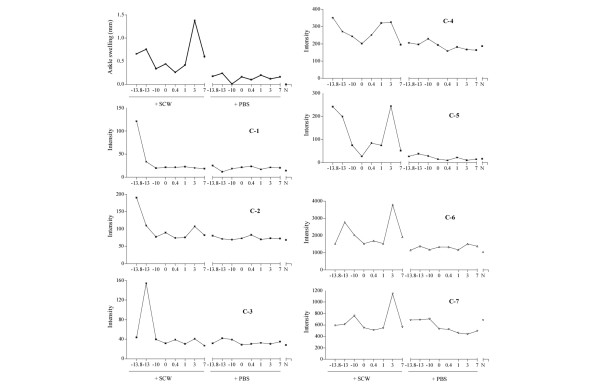
Temporal gene expression profiles in the reactivation model of streptococcal cell wall (SCW)-induced arthritis in rat identified using Spotfire^® ^profile search analysis. The seven different clusters identified are termed C-1 to C-7. Each graph shows the characteristic pattern of expression throughout the time course of disease for a representative gene from the defined cluster. Results are expressed as the mean of the signal intensity of gene expression for each group (four to five samples/group). The number of the cluster to which each gene belongs is included in Table 2. The time course of inflammation, expressed as change in ankle diameter (mm) relative to the starting diameter, is shown in the upper left panel. N, naïve; PBS, phosphate-buffered saline.

The selected 631 dysregulated genes (*P *< 0.01 and Delta > 1.8) were analyzed using Spotfire^® ^profile search analysis and K-means clustering [15], allowing the identification of different patterns and levels of gene expression throughout the time course of disease. As shown in Fig. 5, the upregulated genes were grouped into seven clusters (C-1 to C-7) according to their kinetics of expression. Thus, all genes exhibiting similar patterns of expression at the analyzed time points were grouped into the same cluster (e.g. C-1 for those genes whose expression reached a peak on day -13.8). These genes were also sorted into three K-means clusters according to their level of expression (low, medium and high). The cluster number to which each gene belongs is summarized in Table2.

Interestingly, the expressions of different markers for cell types associated with the pathogenesis of RA were found to be upregulated throughout the time course of SCW-induced arthritis. These markers were grouped into different clusters as follows: C-2 = CD44 (leucocytes, erythrocytes); C-3 = CD2 (T cell, natural killer [NK] cells), E-selectin (SELE; activated endothelial cells); C-4 = L-selectin (SELL; lymphocytes, monocytes and NK cells); C-5 = CD14 (monocytes), ICAM1 (endothelial cells), α M integrin (ITGAM or CD11b; granulocytes, monocytes, NK cells), P-selectin (SELP; endothelial cells, activated platelets), lipocalin 2 (LCN2; neutrophils); C-6 = CD74 (B cells, monocytes), CD38 (activated T cells, plasma cells), CD8a (cytotoxic/suppressor T cells, NK cells); and C-7 = CD3d (T cells), CD4 (helper–inducer T cells). The different temporal expression of these markers highlights that expression levels for CD3d and CD4 were significantly upregulated only at day 3 after challenge, in contrast to CD2 and E-selectin, whose expression was found to be upregulated only at day -13. The rest of the markers exhibited significant fold changes in gene expression at both phases of disease (4 hours after intra-articular injection of SCW, day -13 and day 3 after challenge), except CD8a, CD74 and CD38, which were found to be upregulated at a later time point in the pre-reactivation phase (day -13). Only CD44 was not found to be upregulated on day 3 after challenge. Lipocalin 2, αM integrin and CD8a exhibited the greatest fold changes in gene expression.

### Functional grouping of dysregulated genes

In order to establish functional annotations for the selected dysregulated genes, the biological processes and molecular functions of the genes were investigated using different databases. This search identified 19 ontologies for the upregulated genes, allowing us to organize them according to their major functions (Table 2 and Fig. 2). Because of space limitations in the manuscript, we could not include all of the upregulated genes in Table 2 and Fig. 2. The genes not included were involved in blood coagulation, catabolism, defence response, G-protein-coupled receptor protein signalling pathways, metabolism and protein modification, or were genes with unknown functions (for more information, please see Additional file 1). A hallmark of RA is infiltration of leucocytes into synovial tissue mediated by a complex network of cytokines, adhesion molecules and chemoattractants [18].

Interestingly, most of the genes exhibiting the greatest fold increase in gene expression (Delta > 5) on days -13.8, -13 or 3 were involved in chemotaxis. These included several CC chemokine ligands (CCLs; CCL20, CCL2 [also called SCYA2 or MCP-1]), CXC chemokine ligands (CXCLs; CXCL2, CXCL6 and GRO1), CC chemokine receptors (CCRs; CCR1, CCR2, CCR5), CXC chemokine receptors (CXCRs; CXCR2) and a recently characterized cytokine called chemokine-like factor 1 [19].

Our results also showed marked upregulation (Delta > 5) for numerous genes that are involved in the immune and/or inflammatory response, such as IL-1β, IL-6, TNF-α, TNFRSF1b, IL-1Rn, NOS2, CD8a, VAV1, LST1 (leukocyte specific transcript 1), LCP2 (lymphocyte cytosolic protein 2), FCGR2 (Fc receptor, IgG, low affinity Iib), PTGES (microsomal prostaglandin E synthase-1) and the major histocompatibility complex (MHC) class Ib gene (RTAW2). Other components of the MHC such as MHC class II (HLA-DMA and HLA-DMB) and MHC class Ib RT1.S3 genes were also found to be upregulated in this model. Genes participating in cell adhesion such as TNFIP6, FCNB (ficolin B), CSPG2 (versican), ICAM1 and αM integrin (ITGAM) also exhibited a significant fold increase in gene expression (Delta > 5). Among other genes, some mediators controlling extracellular matrix (ECM) turnover and breakdown under normal and disease conditions, including five matrix metalloproteinases (MMPs; MMP-3, -12, -13, -14 and -23a), the aggrecanase ADAMTS-1, tissue inhibitor of metalloproteinases (TIMP)1, and the secretory leucocyte protease inhibitor (SLPI) were also found to be significantly upregulated in arthritic joints. The majority of the downregulated genes were associated with regulation of metabolism, myogenesis, or regulation of muscle development and transport (Fig. 3).

### Differentially expressed genes: QTL association

From the 441 selected genes that were upregulated during SCW-induced arthritis, we selected a list of 20 genes that exhibited a greater than fivefold change in gene expression and that had not previously been linked to autoimmune arthritis. To further investigate the possibility that these genes play a role in arthritis pathogenesis, their rat chromosomal locations and the locations of their human orthologues were identified and compared with those of rat and human QTLs for autoimmune diseases. Interestingly, 10 of these genes were found to be located in chromosomal regions that mapped to rat and/or human QTLs previously reported to be associated with inflammation, arthritis, or autoimmune diseases, such as systemic lupus erythematosus, multiple sclerosis, allergic rhinitis and asthma (Table 3).

### Analysis of expression profiles of specific transcripts

**Figure 6:**
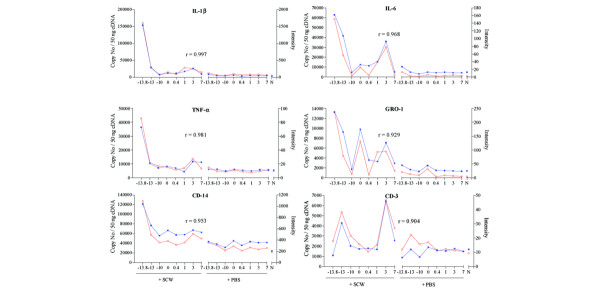
Confirmation of the expression levels of six of the highly differentially expressed genes highlighted in Table 2 by real-time RT-PCR TaqMan^® ^analysis. The graphs compare the gene expression profiles for IL-1β, tumour necrosis factor (TNF)-α, IL-6, GRO1, CD14 and CD3 obtained using two different methods: Affymetrix Rat Genome RAE230A GeneChip^® ^(filled squares) and real-time RT-PCR TaqMan^® ^analysis (open squares). Data are expressed as the mean of signal intensity or the mean of copy no./50 ng cDNA normalized against the housekeeping gene ubiquitin, for all of the samples from the same group (four to five). The Pearson product moment correlation coefficient (r) for each comparison is given. PBS, phosphate-buffered saline; SCW, streptococcal cell wall.

In order to validate microarray data, mRNA expression levels for a subset of genes were quantified by real-time RT-PCR TaqMan^® ^analysis. As shown in Fig. 6, there was a significant correlation (Pearson product moment correlation coefficient r > 0.9 and *P *< 0.01) between the gene expression profiles for the proinflammatory cytokines IL-1β, TNF-α and IL-6, the chemokine GRO1 and the cell markers CD14 and CD3, when microarray data were compared with RT-PCR TaqMan^® ^data. Although the fold changes in gene expression calculated using data from both methods were not exactly the same (probably due to differences in the sensitivities of the assays), the quantitative real-time RT-PCR TaqMan^® ^method verified the results of the gene array analysis.

## Discussion

The temporal expression of multiple disease-associated genes with potential pathophysiological roles in the reactivation model of SCW-induced arthritis in Lewis (LEW/N) rat has not previously been fully addressed. The present study analyzed gene expression profiles in rat joints with SCW-induced arthritis using RAE230A GeneChip^® ^oligonucleotide microarray (Affymetrix Inc.). We chose to profile gene expression in whole ankle joint tissues, which comprises heterogeneous cell types, with the aim of gaining a global insight into the molecular changes associated with arthritis pathology in this model. Analysis of the time course data generated by microarray identified 631 genes (441 upregulated and 190 downregulated) with full-length sequences in databases that were significantly differentially expressed (Delta > 1.8 and *P *< 0.01). Our experimental design (time course study) and use of K-means cluster analysis allowed us to identify specific patterns of gene expression for the different dysregulated genes, highlighting the importance of performing kinetic studies to identify the time point at which a particular gene is maximally expressed. Thus, these gene expression data indicate optimal times for measuring potential disease biomarkers in rat joints, and our approach offers a useful tool with which to investigate the clinical efficacy and mechanism of action of novel therapeutic agents in rat SCW-induced arthritis.

Changes in gene expression may reflect regulation at the mRNA level or changes in the number of cells (proliferation or infiltration) that synthesize these mRNAs. Thus, optimally, microarray analysis should be conducted in isolated populations of cells so that differential gene expression may be directly correlated with transcription of the genes. However, complex diseases such as RA involve extensive tissue injury, and not all of the cell types that contribute to RA pathogenesis have been identified. Hence, analysis of the damaged tissue, rather than analysis of an isolated cell type, increases the probability that differential gene expression will be examined in those cells that are important in RA pathogenesis. In the present study we conducted a global analysis of coordinated gene expression in injured tissue. Further bioinformatic analysis of the data to examine cell markers, and genes whose expression may correlate with them, in combination with analysis of the cell populations present in the arthritic joint using immunohistochemistry or fluorescence activated cell sorting techniques, would be required to corroborate the differential gene expression of a particular gene of interest. Previous studies have already shown that cell-specific gene expression patterns can indicate the presence of immune cells [20]. RAE230A GeneChip^® ^oligonucleotide microarray analysis identified the expression of different markers for cell types associated with the pathogenesis of RA. Based on the level of gene expression and Delta values detected for the different markers, our results suggest that the main cell types present in arthritic joints in this model are T cells, neutrophils, monocytes/macrophages and B cells, confirming previous descriptions of the joint cell composition in this model [6,21].

Gene expression profiling of arthritic rat joints revealed a spectrum of genes exhibiting extensive inflammatory activity, infiltration of activated cells, angiogenesis, regulation of apoptosis and ECM remodelling activities. Most of the genes found to be upregulated in SCW-induced arthritic joints have also been reported to be highly expressed in human RA synovial tissue [22,23] or in joints from other rodent experimental arthritis models [10,11,24,25]. The upregulated expression of TNF-α, IL-1α, IL-1β, IL-4R, P-selectin, MIP-1α (CCL3), MCP-1 (CCL2), NOS2 and NOS3 [6-8] demonstrated in the present study is in agreement with previous observations of the dependency of the rat SCW-induced arthritis model on these mediators. The SLPI has previously been reported to be upregulated in arthritic joints and to mediate tissue destruction and inflammation in a rat model of arthritis induced by intraperitoneal injection of SCW [26]. Similar results were found in our study, because significant upregulation of SLPI gene expression was observed during both phases of the disease. Additionally, previous studies have shown that nuclear factor-κB (NF-κB) is activated in the synovium of rats with SCW-induced arthritis and that inhibition of the activity of this transcription factor enhances synovial apoptosis, which is consistent with the potential involvement of NF-κB in synovial hyperplasia [27]. In accord with these observations, the microarray data showed early upregulation of genes involved in the NF-κB signalling pathway, such as NF-κB1 (p50 or p105), NFKBIA (IκBα), TNF-α, TNFRSF1a and TNFRSF1b, suggesting a possible regulatory role of NF-κB in the transcription of genes that mediate disease progression in SCW-induced arthritis.

Histopathological studies in arthritic rat joints from the reactivation SCW-induced arthritis model have shown that only moderate histological changes in articular cartilage, with few erosive effects on bone, occur at early stages in the flare reaction (day 3), whereas evident cartilage degradation is observed at later time points (20 days after intravenous challenge with SCW) [4]. The microarray data suggest that tissue remodelling is an active process in this model because abundant expression of collagen-related genes (Col5A2, Col5A3, Col12A1 and Col18A1), enzymes that degrade matrix molecules such as MMPs and the aggrecanase ADAMTS-1 (a disintegrin-like and metalloproteinase with thrombospondin type 1 motif, which is capable of cleaving versican), together with other genes that control ECM turnover and breakdown (TIMP1, PLAU [plasminogen activator, urokinase], PLAU receptor [PLAUR]), were found to be upregulated in arthritic joints. MMP-3 (stromelysin) appears to be pivotal in the activation of collagenases, whereas MMP-13 is crucial in collagen breakdown [28]. The PLAU/PLAUR system plays a critical role in cartilage degradation during osteoarthris by regulating pericellular proteolysis mediated by serine proteases [22,29]. The complement system has also been reported to participate in tissue injury during inflammatory and autoimmune diseases [30], and ficolins can initiate the lectin pathway of complement activation through attached serine proteases (Mannan-binding lectin serine proteases [MASPs]) [31]. Interestingly, the microarray data revealed significant upregulation of the first complement component C1, which exerts collagenolytic activity in addition to the role it plays in the classic cascade [29]. In addition, upregulation of the expression of C2, C3, ficolin B (FCNB) and MASP1 was also noted, supporting the concept that activation of the complement system, together with the imbalance between MMPs, TIMPs and other related molecules, could mediate cartilage destruction in this experimental model of RA.

In our analysis we also identified 10 genes that are differentially expressed in arthritic joints and that that map to genomic regions previously reported to be QTLs for autoimmune diseases. Although it is premature to suggest that the 10 genes are candidates for these QTLs, our observations suggest that expression of these genes may influence the onset, severity and/or susceptibility to arthritis in this animal model. Of particular interest is KDAP (napsin) because of the high fold increase in gene expression observed in arthritic joints from SCW-injected animals (D = 48.2 on day 3). This aspartic protease was shown to be expressed in kidney, lung and lymphoid organs of mice [32], and it has been suggested that it functions as a lysosomal protease involved in protein catabolism in renal proximal tubules [33]. However, little is known about the role of KDAP in other organs and tissues. Interestingly, human KDAP resides on chromosome 19q13.3–19q13.4, a region previously identified to be involved in susceptibility to autoimmune diseases, including systemic lupus erythematosus, multiple sclerosis and insulin-dependent diabetes mellitus [34,35]. Our results show, for the first time, that KDAP gene expression is upregulated in experimental arthritis tissue, and suggest that further characterization is required to unravel the biological/pathological activities of this gene in RA.

The microarray data also revealed high upregulation in runt-related transcription factor 1 (RUNX1) and a group of transporter genes (SLC11A1, SLC13A3, SLC1A3, SLC21A2 [MATR1], SLC28A2, SLC29A3, SLC5A2 and SLC7A7), from which the prostaglandin transporter gene MATR1 exhibited the greatest upregulation on day 3 after intravenous challenge with SCW. The rat MATR1 gene maps to the type II collagen induced arthritis severity QTL6 (Cia6) [36], and its human orthologue is located within autoimmune disease QTLs for asthma, psoriasis and atopic dermatitis [37-39]. Several authors reported linkage of SLC11A1 (also named NRAMP1) to human RA [40-42]. The Z-DNA forming polymorphic repeat in the RUNX1-containing promoter region of human SLC11A1 may contribute to the differing allelic associations observed with infectious versus autoimmune disease susceptibility [43]. Recent studies reported that regulation of expression of organic cation transporter gene SLC22A4 by RUNX1 is associated with susceptibility to RA [44]. Other transporter genes (SLC12A8 and SLC9A3R1) have also been linked to susceptibility to other autoimmune diseases such as psoriasis [45]. These observations together suggest that RUNX1 and the transporter genes found to be differentially expressed in arthritic joints may contribute to arthritis susceptibility and to the inflammatory processes that mediate the pathology of this model.

## Conclusion

The present study identified the temporal gene expression profiles of hundreds of genes, including cytokines, chemokines, adhesion molecules, transcription factors, apoptotic and angiogenesis mediators, whose expression is associated with onset and progression of arthritis pathology in rat joints from the reactivation model of SCW-induced arthritis in Lewis (LEW/N) rat. This transcript profiling offers not only the optimal kinetics of expression for different potential disease biomarkers, but it also improves our understanding of the molecular events that underlie the pathology in this animal model of RA. In addition, although the majority of genes found to be differentially expressed in this model were previously associated with human RA, further genes not previously linked to autoimmune diseases were identified, providing a resource for future research and for the development of new therapeutic targets.

## Abbreviations

ANOVA = analysis of variance; CCL = CC chemokine ligand; CCR = CC chemokine receptor; CXCL = CXC chemokine ligand; CXCR = CXC chemokine receptor; ECM = extracellular matrix; EST = expressed sequence tag; IL = interleukin; MCP = monocyte chemoattractant protein; MHC = major histocompatibility complex; MIP = macrophage inflammatory protein; MMP = matrix metalloproteinase; NF-κB = nuclear factor-κB; NK = natural killer; NOS = nitric oxide synthase; PBS = phosphate-buffered saline; PCA = principal component analysis; PCR = polymerase chain reaction; PG-PS = peptidoglycan–polysaccharide; QTL = quantitative trait locus; RA = rheumatoid arthritis; RT = reverse transcription; SCW = streptococcal cell wall; SLPI = secretory leucocyte protease inhibitor; TIMP = tissue inhibitor of matrix metalloproteinase; TNF = tumour necrosis factor.

## Competing interests

The author(s) declare that they have no competing interests.

## Authors' contributions

RI carried out the study design, *in vivo *experiments, total RNA extractions, RT-PCR analysis of data and manuscript preparation. CC and SG performed the microarray experiments and statistical analysis of the array data. MD and PL carried out the study design and collaborated in the preparation of the manuscript.

## Supplementary Material

Additional File 1 Excel spreadsheets summarizing all of the genes upregulated (Delta > 1.8 and *P *< 0.01) and downregulated (Delta < 1.8 and *P *< 0.01) in ankle joints from SCW-induced arthritis in Lewis (LEW/N) rats on days -13.8 (4 hours after intra-articular injection of SCW), -13 and 3. Data are expressed as the mean fold increase in gene expression (D = Delta) in SCW-injected animals compared with the expression in the corresponding PBS control group, along with *P *values. Click here for file

## Figures and Tables

**Table 1:** TaqMan^® ^probes and primers for the genes of interest

Gene of interest	Forward primer	Reverse primer	Probe
IL-1β	5'-CACCTCTCAAGCAGAGCACAG	5'-GGGTTCCATGGTGAAGTCAAC	5'-6-FAM-TGTCCCGACCATTGCTGTTTCCTAGG-TAMRA
IL-6	5'-CAAGACCATCCAACTCATCTTG	5'-CACAGTGAGGAATGTCCACAAAC	5'-6-FAM-TCGGCAAACCTAGTGTGCTATGCCTAAGCA-TAMRA
TNF-α	5'-CCAGGTTCTCTTCAAGGGACAA	5'-CTCCTGGTATGAAATGGCAAATC	5'-6-FAM-CCCGACTATGTGCTCCTCACCCACA-TAMRA
GRO1	5'-TGTGTTGAAGCTTCCCTTGGA	5'-TGAGACGAGAAGGAGCATTGGT	5'-6-FAM-TGTCTAGTTTGTAGGGCACAATGCCCT-TAMRA
CD14	5'-GGACGAGGAAAGTGTCCGCT	5'-AGGTACTCCAGGCTGCGACC	5'-6-FAM-TTCTATGCGCGGGGGCGGAA-TAMRA
CD3	5'-GGATGGAGTTCGCCAGTCAA	5'-GGTTTCCTTGGAGACGGCTG	5'-6-FAM-ACAGGTCTACCAGCCCCTCAAGGACCG-TAMRA
Ubiquitin	5'-CGAGAACGTGAAGGCCAAGA	5'-GGAGGACAAGGTGCAGGGTT	5'-6-FAM-CCCCTGACCAGCAGAGGCTCATCTTTG-TAMRA

IL, interleukin; TNF, tumour necrosis factor.

**Table 2:** Genes upregulated in ankle joints from SCW-induced arthritis in Lewis (LEW/N) rats

Accession no.	Gene name	Day -13.8		Day -13		Day 3		C	I
				
		Delta	*P*	Delta	*P*	Delta	*P*		
Angiogenesis									
NM_030985	AGTR1	_	_	_	_	4.0	2.3E-05	7	L
AI639162	ANGPT1	_	_	_	_	1.8	8.5E-08	7	L
NM_031012	ANPEP	_	_	_	_	2.2	2.9E-18	7	M
AI101782	COL18A1	_	_	_	_	2.9	6.3E-26	7	L
AI170324	FIGF	_	_	1.6	9.4E-04	2.4	4.6E-11	6	L
NM_012620	SERPINE1	6.6	2.3E-06	_	_	27.6	0.0E+00	4	L

Cell adhesion									
NM_012830	CD2	_	_	2.1	2.9E-04	_	_	3	L
NM_054001	CD36L2	_	_	_	_	2.6	5.4E-05	7	L
AF065147	CD44	2.1	2.1E-08	1.6	1.1E-03	_	_	2	M
BE108345	COL12A1	_	_	_	_	2.5	4.6E-43	7	M
AI172281	COL5A2	_	_	_	_	2.0	9.3E-06	7	H
NM_021760	COL5A3	_	_	_	_	3.1	6.9E-27	7	M
AF084544	CSPG2	2.3	2.4E-03	2.1	5.8E-04	8.9	7.2E-37	5	L
NM_053719	EMB	_	_	_	_	3.5	1.7E-19	7	L
NM_053634	FCNB	8.7	3.0E-28	8.0	1.1E-17	29.0	0.0E+00	5	M
AI236745	GALNT1	_	_	_	_	2.9	0.0E+00	7	L
NM_133298	GPNMB	_	_	2.2	3.6E-12	2.5	0.0E+00	6	H
NM_012967	ICAM1	8.9	0.0E+00	4.4	7.2E-09	4.0	2.2E-11	5	L
AF268593	ITGAM	2.0	2.6E-03	3.8	1.7E-15	6.4	3.3E-18	5	L
BI296880	ITGB3	1.5	7.0E-03	_	_	2.1	2.3E-03	4	L
AF003598	ITGB7	2.0	8.9E-14	1.6	1.2E-03	1.9	1.1E-06	5	L
U56936	KLRB1B	_	_	_	_	3.0	6.4E-03	7	L
NM_022393	MGL	_	_	2.1	7.3E-08	2.1	2.3E-11	6	L
U72660	NINJ1	_	_	_	_	1.8	6.6E-28	7	M
BE097805	PCDHGC3	_	_	1.8	3.0E-03	_	_	3	L
AJ299017	RET	_	_	_	_	2.8	1.6E-08	7	L
AF071495	SCARB1	_	_	_	_	1.8	6.9E-03	7	L
L25527	SELE	_	_	3.1	3.6E-04	_	_	3	L
D10831	SELL	1.6	9.3E-05	_	_	1.8	6.9E-06	4	L
BI296054	SELP	1.8	7.1E-08	1.9	2.6E-07	2.2	4.2E-13	5	L
AI176034	TNC	_	_	_	_	2.6	3.7E-26	7	M
AF159103	TNFIP6	2.2	6.2E-04	2.2	3.1E-05	5.0	1.3E-21	5	L
NM_031590	WISP2	_	_	_	_	2.6	0.0E+00	7	M

Chemotaxis									
NM_053619	C5AR1	1.6	5.0E-06	2.4	9.6E-21	2.7	2.8E-32	5	M
NM_019205	CCL11	3.9	2.3E-03	3.7	2.3E-07	_	_	2	L
NM_057151	CCL17	2.2	1.1E-05	_	_	_	_	1	L
AF053312	CCL20	8.3	7.3E-19	10.2	2.8E-10	15.5	3.5E-32	5	L
U22414	CCL3	15.3	1.7E-19	3.2	3.8E-05	2.1	1.1E-08	5	L
U06434	CCL4	6.0	1.5E-17	_	_	_	_	1	L
NM_031116	CCL5	_	_	2.6	5.9E-11	2.0	1.1E-03	6	L
NM_020542	CCR1	5.2	1.4E-15	2.1	3.0E-05	2.1	3.2E-03	5	L
NM_021866	CCR2	5.1	2.7E-07	3.3	2.9E-05	6.9	2.7E-13	5	L
NM_053960	CCR5	6.2	4.3E-19	6.0	1.8E-09	6.0	1.4E-10	5	L
D87927	CINC2	3.5	7.0E-03	_	_	_	_	1	L
AF253065	CKLF1	3.3	6.3E-09	3.0	2.7E-07	8.2	8.6E-08	5	L
NM_022218	CMKLR1	_	_	2.5	3.4E-03	_	_	3	L
U22520	CXCL10	3.2	4.4E-09	2.5	9.0E-03	1.4	1.3E-03	5	L
NM_053647	CXCL2	38.7	1.6E-07	2.3	9.1E-03	2.6	1.0E-03	5	L
NM_022214	CXCL6	2.2	2.3E-04	_	_	7.5	3.2E-06	4	L
NM_017183	CXCR2	10.6	1.5E-07	3.6	1.3E-03	_	_	2	L
NM_053415	CXCR3_V1	_	_	_	_	1.9	9.5E-04	7	L
AA945737	CXCR4	1.6	1.7E-03	1.7	3.9E-04	3.4	2.7E-15	5	L
NM_030845.1	GRO	17.1	0.0E+00	23.0	2.4E-04	19.8	1.8E-12	5	L
NM_053321	PTAFR	_	_	2.5	2.0E-03	_	_	3	L
NM_031530	SCYA2	3.4	6.0E-26	3.2	1.8E-16	6.0	0.0E+00	5	M

Complement activation									
D88250	C1S	_	_	1.6	4.4E-03	1.8	7.5E-22	6	M
_	C2	6.9	9.20E-42	3.5	1.28E-11	16.8	0.0E+00	5	L
NM_016994.1	C3	2.7	2.0E-10	3.0	5.4E-12	10.4	0.0E+00	5	L
AI169829	MASP1	_	_	_	_	2.4	8.5E-08	7	L

Immune response/inflammatory response									
XM_215303	RT1.S3	_	_	2.0	0.0012	1.6	1.5E-03	6	L
AF307302	BTNL2	_	_	2.1	1.0E-15	3.2	0.0E+00	6	M
NM_021744	CD14	2.8	7.8E-18	2.0	4.4E-06	1.7	7.3E-05	5	M
NM_012705	CD4	_	_	_	_	1.8	1.3E-07	7	L
NM_013069	CD74	_	_	2.2	3.5E-18	2.7	1.1E-31	6	H
NM_031538	CD8a	_	_	9.5	2.7E-03	10.9	6.2E-07	6	L
BI282755	EDG3	_	_	_	_	2.1	5.9E-03	7	L
X73371	FCGR2	3.1	1.4E-20	3.8	2.4E-08	6.5	0.0E+00	5	L
NM_053843	FCGR3	2.2	3.3E-15	2.0	2.8E-12	2.6	0.0E+00	5	M
NM_133624	GBP2	3.4	6.2E-35	_	_	1.5	3.8E-06	4	L
AF176534	HFE	_	_	_	_	1.8	6.6E-03	7	L
XM_215347	HLA-DMA	_	_	2.0	1.22E-14	2.7	0.0E+00	6	L
_	HLA-DMB	_	_	2.0	5.42E-15	3.2	1.1E-13	6	M
NM_022605	HPSE	_	_	_	_	1.9	3.0E-09	7	L
NM_133533	IGB	_	_	_	_	2.9	8.4E-09	7	L
NM_053374	IGIFBP	_	_	2.2	7.2E-03	_	_	3	L
AJ245643	IL1a	2.7	1.2E-03	_	_	_	_	1	L
NM_031512	IL1b	22.0	1.1E-30	9.5	4.5E-15	5.7	1.6E-35	5	L
NM_053953	IL1R2	2.5	1.9E-15	_	_	_	_	1	L
NM_022194	IL1RN	7.4	2.8E-03	_	_	_	_	1	L
NM_012589	IL6	10.0	7.3E-17	20.7	7.9E-04	21.4	5.5E-17	5	L
NM_013110	IL7	_	_	_	_	2.8	2.4E-04	7	L
NM_012591	IRF1	2.9	4.7E-13	2.6	2.8E-08	3.3	2.0E-13	5	L
NM_130741	LCN2	2.4	1.7E-09	3.4	9.8E-12	13.2	0.0E+00	5	M
BF282471	LCP2	2.6	2.2E-04	3.3	2.4E-04	6.2	4.4E-06	5	L
NM_022634	LST1	4.9	6.2E-14	6.3	6.4E-14	16.7	2.0E-36	5	L
NM_031634	MEFV	2.7	1.9E-07	_	_	1.9	7.4E-05	4	L
X52711	MX1	_	_	2.8	4.9E-07	1.9	2.1E-15	6	L
NM_134350	MX2	_	_	2.9	5.9E-04	_	_	3	L
NM_053734	NCF1	_	_	2.0	9.4E-16	2.0	3.7E-06	6	L
AA858801	NFKB1	2.1	1.1E-12	_	_	_	_	1	M
AW672589	NFKBIA	2.5	5.2E-36	_	_	_	_	1	M
L12562	NOS2A	6.0	1.9E-05	_	_	_	_	1	L
Z18877	OAS1	1.6	8.4E-06	2.4	3.8E-06	1.8	3.7E-06	5	L
NM_053288	ORM1	_	_	2.0	7.0E-04	3.1	9.8E-19	6	L
NM_031713	PIRB	2.4	3.9E-06	2.5	2.6E-06	3.3	7.6E-10	5	L
AF349115	PPBP	_	_	_	_	3.2	8.5E-03	7	L
NM_080767	PSMB8	1.5	3.0E-03	2.3	2.0E-09	3.3	0.0E+00	5	L
AI599350	PSMB9	2.0	2.0E-07	2.1	7.4E-09	3.7	2.3E-25	5	L
AB048730	PTGES	8.2	8.1E-40	3.9	1.0E-04	2.4	6.7E-04	5	L
NM_012645	RT1Aw2	_	_	3.3	0.000334	5.4	2.7E-10	6	L
X57523.1	TAP1	1.6	2.8E-04	1.6	9.8E-03	2.4	5.6E-07	5	L
NM_021578	TGFB1	_	_	2.1	8.4E-06	2.6	1.7E-10	6	L
AA819227	TNF	11.1	1.3E-27	2.5	1.9E-04	_	_	2	L
BM390522	TNFRSF1b	14.3	8.2E-19	3.7	4.2E-06	8.0	3.5E-06	5	L
NM_012759	VAV1	4.6	7.1E-05	7.6	1.2E-07	10.8	1.2E-12	5	L

Proteolysis and peptidolysis									
NM_024400	ADAMTS1	3.1	9.2E-16	2.1	7.0E-04	3.5	1.3E-16	5	L
AA849399	CTSZ	1.6	6.4E-08	1.5	8.9E-12	3.4	1.6E-33	5	M
NM_012582	HP	2.1	4.8E-20	_	_	1.7	5.5E-05	4	L
NM_031670	KDAP	18.8	8.7E-23	6.6	5.0E-07	48.2	2.3E-37	5	L
AF154349	LGMN	_	_	2.1	1.8E-06	2.8	0.0E+00	6	M
NM_053963	MMP12	_	_	4.1	8.6E-05	7.7	8.2E-13	6	L
M60616.1	MMP13	_	_	_	_	2.0	4.7E-08	7	M
X83537	MMP14	_	_	_	_	1.8	2.1E-17	7	H
NM_053606	MMP23A	_	_	_	_	2.1	1.6E-11	7	L
NM_133523	MMP3	2.9	5.7E-29	2.7	1.4E-12	9.3	0.0E+00	5	H
AI102069	NSF	_	_	1.7	8.1E-03	1.8	3.9E-04	6	L
BF549923	PCSK5	_	_	1.8	1.5E-03	3.4	7.5E-21	6	L
X63434	PLAU	_	_	_	_	1.8	2.9E-14	7	M
AF007789	PLAUR	6.2	4.6E-04	_	_	4.9	7.2E-03	4	L
NM_053372	SLPI	2.6	8.3E-09	2.6	5.4E-22	7.0	0.0E+00	5	M
NM_053819	TIMP1	2.2	0.0E+00	1.8	5.9E-09	6.4	0.0E+00	5	H
NM_053299	UBD	_	_	_	_	4.7	9.1E-04	7	L

Signal transduction									
NM_019285	ADCY4	_	_	_	_	2.3	3.5E-05	7	L
BF285345	ARRB2	_	_	1.8	2.5E-05	2.5	6.5E-20	6	L
NM_057196	BAIAP2	_	_	4.4	5.5E-03	_	_	3	L
NM_012766	CCND3	_	_	_	_	2.1	6.0E-08	7	L
NM_013169	CD3d	_	_	_	_	3.9	3.7E-07	7	L
AF065161	CISH	2.5	1.1E-03	_	_	_	_	1	L
NM_031352	DBNL	_	_	1.7	6.6E-11	1.8	1.2E-04	6	L
BI278868	EPIM	_	_	_	_	2.1	6.7E-03	7	L
NM_024147	EVL	_	_	_	_	3.6	8.0E-09	7	L
L02530	FZD2	_	_	_	_	3.3	6.3E-07	7	L
NM_030829.1	GPRK5	_	_	_	_	2.0	1.0E-04	7	L
U87863.1	HGS	_	_	1.9	3.6E-03	_	_	3	L
AY044251	IL13RA1	_	_	_	_	3.2	3.0E-08	7	L
AI178808	IL2RG	2.4	8.1E-14	2.5	10.0E-23	5.6	0.0E+00	5	L
NM_133380	IL4R	_	_	6.5	4.8E-05	7.5	3.4E-18	6	L
NM_017020	IL6R	_	_	1.8	2.4E-10	1.8	1.6E-12	6	L
NM_019311	INPP5D	_	_	_	_	1.9	7.9E-20	7	L
NM_012798	MAL	2.1	1.9E-04	_	_	_	_	1	L
AW533194	MAPK10	2.4	5.7E-03	_	_	_	_	1	L
AF411318	MT1A	2.6	2.4E-27	2.4	2.2E-04	3.4	6.0E-34	5	M
NM_012613	NPR1	_	_	_	_	3.3	1.9E-03	7	L
U32497	P2RX4	_	_	1.6	4.2E-15	1.9	2.5E-20	6	L
AF202733	PDE4B	2.4	1.1E-07	2.5	8.1E-04	2.3	2.5E-03	5	L
BE099769	PLAA	_	_	2.5	8.7E-03	_	_	3	L
X04440	PRKCB1	_	_	_	_	1.8	3.3E-08	7	L
AF254800	RAB0	_	_	_	_	1.9	7.8E-04	7	L
NM_019250	RALGDS	1.9	8.1E-05	_	_	_	_	1	L
NM_021661	RGS19	_	_	_	_	1.8	1.8E-05	7	L
AF321837	RGS2	_	_	_	_	2.3	2.4E-09	7	L
NM_053338	RRAD	7.0	5.2E-05	4.8	1.6E-05	4.0	6.5E-03	5	L
BE117558	SFRP1	_	_	_	_	1.8	2.4E-08	7	M
BF389682	SOCS3	3.8	0.0E+00	2.0	1.2E-05	3.6	3.2E-33	5	L
NM_022230	STC2	_	_	3.1	2.2E-03	_	_	3	L
BG668493	STMN2	_	_	2.3	2.6E-06	14.0	7.2E-42	6	L
U21683	SYK	_	_	_	_	1.8	2.1E-05	7	L

Genes upregulated (Delta > 1.8 and *P *< 0.01) on days -13.8 (4 hours after intra-articular injection of streptococcal cell wall [SCW]), -13 and 3 are grouped by their general ontology and clustered based on their similarity in terms of pattern of expression (C) and expression level (I). Data are expressed as the mean fold increase in gene expression (Delta) in SCW-injected animals as compared with expression in the corresponding phosphate-buffered saline (PBS) control group (four to five animals/group), along with the *P *value. C, number of clusters to which the gene corresponds (trend plots are given in Fig. 6); I, intensity of gene expression (L = low intensity [0–500], M = medium intensity [500–1500], H = high intensity [1500–4000]). A line (_) in the Delta or *P *cell indicates that the gene was not found to be differentially expressed at that particular time point.

**Table 3:** Upregulated genes (Delta > 5, *P *< 0.01) not previously reported to be associated with arthritis

Accession no.	Gene	Delta	Rat CL	Rat QTLs	Human CL	Human QTLs
NM_178144	AMIGO3	Nd/Nd/5.9	8q32	Cia6	3p21.31	Asthma
NM_130411	CORO1A	3.1/2.7/6.6	1q36	Pia11	16p12.1	Blau syndrome, asthma
NM_024381	GYK	6.7/Nd/Nd	Xq22	Cia19	Xp21.3	Allergic rhinitis
NM_031670	KDAP	18.8/6.6/48.2	1q22	_	19q13.3	Asthma, SLE, MS, SD
NM_569105	LCP2	2.6/3.3/6.2	10q12	Cia16, Pia15	5q33.1	RA, PDB, asthma, IBD, psoriasis, ATD
NM_021586	LTBP2	Nd/Nd/6.5	6q31	Pia3, Pia24	14q24	SLE, MODY3
NM_198746	Ly-49.9	Nd/2.0/5.6	4q42	Cia13, Cia24, Pia7, Pia23, Oia2, Oia7, Oia8, Ciaa4	12p13-p12	RA, allergic rhinitis, hypophosphataemic rickets
NM_022667	MATR1	1.7/1.9/5.7	8q32	Cia6	3q21	Atopic dermatitis, asthma, psoriasis
NM_133306	OLR1	8.3/2.8/3.7	4q42	Cia13, Cia24, Pia7, Pia23, Oia2, Oia7, Oia8, Ciaa4	12p13.2-p12.3	RA, hypophosphataemic rickets, allergic rhinitis
NM_053687	SLFN4	5.8/4.6/4.8	10q26	Cia16, Cia21, Cia22, Cia23, Oia4, Ciaa2	17q11.2-q21.1	SLE, MS

The rat chromosomal location and the chromosomal locations of the corresponding human orthologue were identified and mapped to quantitative trait loci (QTLs) previously associated with inflammation, arthritis and/or other autoimmune diseases. Delta values are given for the following time points: day -13.8/day -13/day 3. ATD, autoimmune thyroid disease; CIA, type II collagen-induced arthritis; Ciaa, CIA autoantibody; CL, chromosome location; IBD, inflammatory bowel disease; MOYD 3, maturity-onset diabetes of the young 3; MS, multiple sclerosis; Nd, not differentially expressed; Oia, oil-induced arthritis; PDB, Paget's disease of bone; PIA, pristane-induced arthritis; RA, rheumatoid arthritis; SD, spondylocostal dysostosis; SLE, systemic lupus erythematosus.
